# Supplementary material for: Neurofilament light chain as a potential biomarker for monitoring neurodegeneration in X-linked adrenoleukodystrophy
Source: Nat Commun. 2021 Mar 22;12:1816. doi: 10.1038/s41467-021-22114-2 (PMC7985512; doi:10.1038/s41467-021-22114-2)
Supplement: Supplementary file 1 — Supplementary Information [file 41467_2021_22114_MOESM1_ESM.pdf]

ELECTRONIC SUPPLEMENTARY MATERIAL

Weinhofer et al., „Neurofilament light chain as a potential biomarker for monitoring neurodegeneration in X-linked adrenoleukodystrophy"

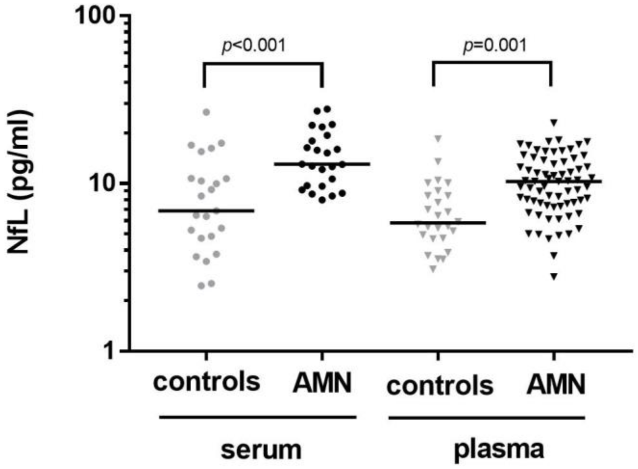

Supplementary Figure 1. NfL in plasma and serum samples of AMN patients. The AMN and adult healthy control sample sets were subdivided into plasma (AMN: *n*=50, total sample number=69, median age=40 years, median NfL=10.3 [7.5-13.6]; control: *n*=25, total sample number=26, median age=34 years, median NfL=5.8 [4.7-8.6]) and serum samples (AMN: *n*=14, total sample number=24, median age=43 years, median NfL=13.1 [9.7-19.0]; control: *n*=23, total sample number=23, median age=44 years, median NfL=6.9 [4.7-10.7]). Whereas NfL was significantly increased in both serum and plasma samples from AMN patients when compared to healthy controls, these group differences did not differ significantly between sample type (*p*=0.205). The potential influence of sample type (serum vs. plasma) on the difference of NfL between control and AMN measurements was investigated using a mixed model with fixed effects for measurement group (control vs. AMN) and for sample type as well as their interaction and a random ID factor (two-sided tests; no multiplicity adjustment). Source data are provided as a Source Data file.

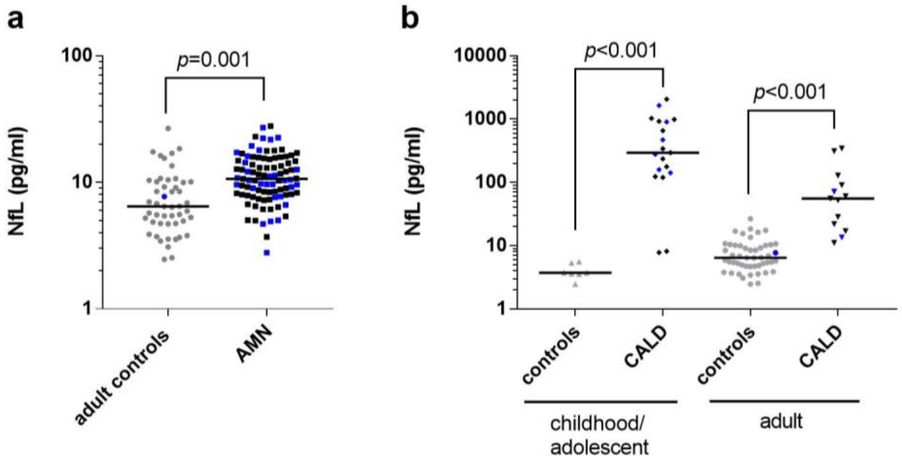

Supplementary Figure 2. Comparison of NfL levels in X-ALD patients and healthy controls of similar age.

a NfL in plasma and serum samples of non-inflammatory AMN patients (*n*=61, median age=40 years, total sample number =93) and healthy adult controls (*n*=48, median age=39 years, total sample number=49). The median NfL level is indicated by a horizontal line. Longitudinal samples are indicated in blue, the latest time point of sampling is marked in grey (healthy controls) and black (X-ALD). Comparison of log(NfL) levels was done using a linear mixed model adjusted for sample type with addition of a random ID factor to account for longitudinal sampling of some individuals (two-sided test). **b** NfL in plasma and serum samples of inflammatory childhood/adolescent CALD (*n*=13, median age=12, total sample number=19), adult CALD patients (*n*=11, median age=44, total sample number=13) and healthy controls of similar age (adults: *n*=48, median age=39, total sample number=49; childhood/adolescent: *n*=7, median age=11, total sample number=7). The median level is indicated by a horizontal line. Total sample numbers include samples obtained longitudinally from the same individuals (healthy controls or patients throughout disease progression. Longitudinal samples are indicated in blue, the latest time point of sampling is marked in grey (healthy controls) and black (X-ALD). Comparison of log(NfL) levels was done using a linear mixed model adjusted for sample type with addition of a random ID factor to account for longitudinal sampling of some individuals (two-sided test). In (b), the magnitude of difference between CALD and controls is significantly different between children/adolescents and adults (*p*=0.005). However, both comparisons within the two age groups are highly significant (both *p*<0.001). The potential influence of age group (children/adolescent vs. adult) on the difference of NfL between control and CALD measurements is investigated using a mixed model with fixed effects for measurement group (control vs. CALD) and for age group as well as their interaction and a

random ID factor (two-sided tests; no multiplicity adjustment). Source data are provided as a Source Data file.

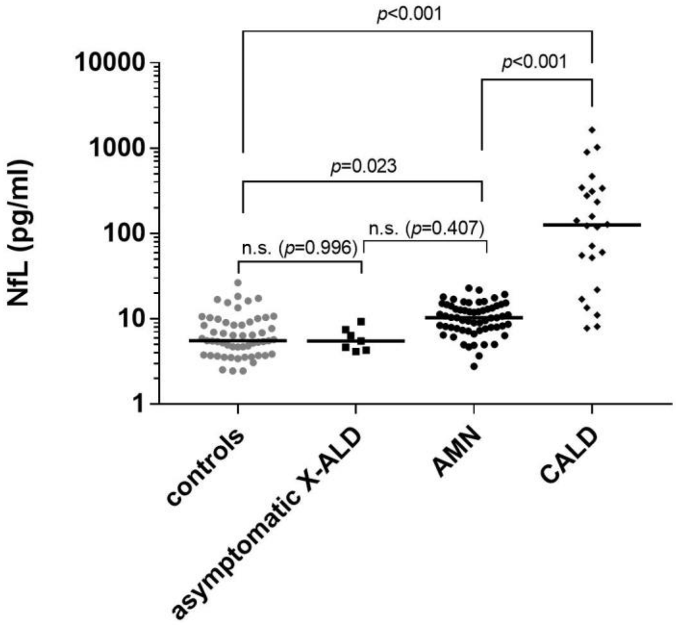

Supplementary Figure 3. NfL levels in X-ALD patients without longitudinal sampling. NfL in plasma and serum samples of asymptomatic X-ALD patients (*n*=7, median age=26), non-inflammatory AMN (*n*=61, median age=42), inflammatory CALD (*n*=24, median age=19) and healthy controls (*n*=55, median age=37). Values represent the first time point of sampling. The median NfL level is indicated by a horizontal line. Comparison of log(NfL) levels was done using a linear mixed model adjusted for sample type (serum vs. plasma). Multiple testing was corrected by Tukey´s method. Source data are provided as a Source Data file.

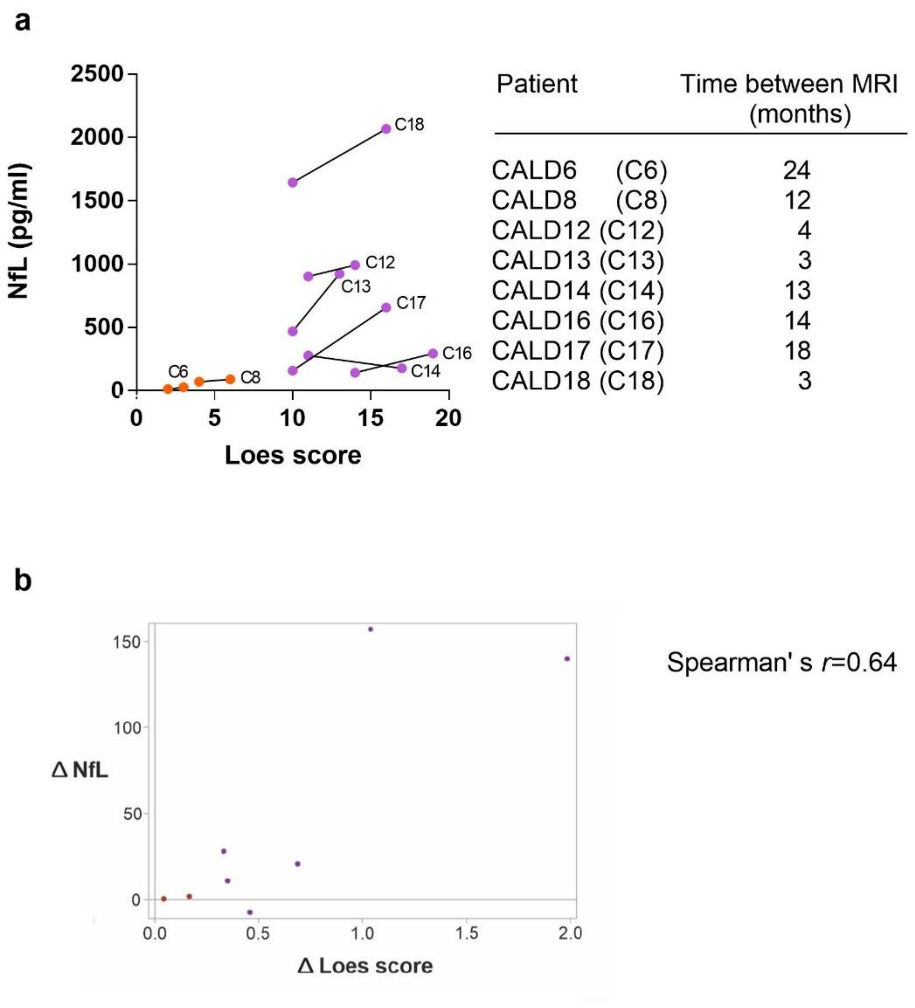

Supplementary Figure 4. Longitudinal analysis of NfL during CALD disease progression. **a** Blood NfL levels in six childhood and adolescent CALD (lilac) and two adult CALD patients (orange) during disease progression over 3 to 24 months, with disease severity scored by MRI according to Loes. **b** Correlation analysis between slopes (change per month) of NfL levels and Loes scores, respectively, over time (Spearman's *r*=0.64). Childhood/adolescent CALD, lilac filled circles; adult CALD, orange filled circles. Source data are provided as a Source Data file.

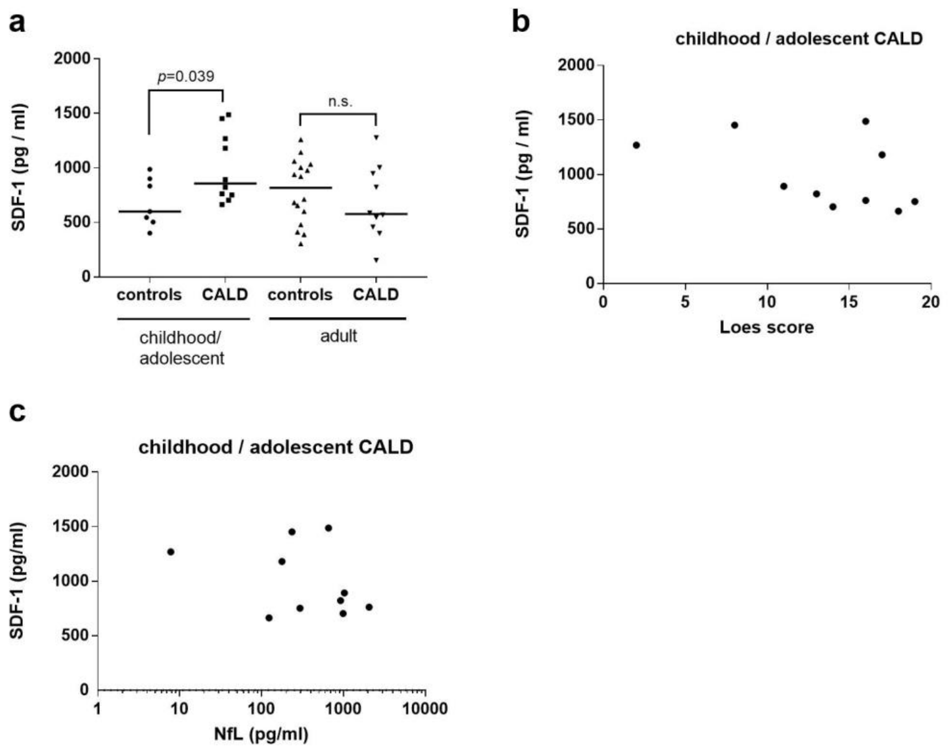

Supplementary Figure 5. No correlation between blood SDF-1 and NfL levels in CALD patients. a SDF-1 levels in childhood/adolescent CALD (*n*=10), adult CALD (*n*=10), childhood/adolescent controls (*n*=7) and adult controls (*n*=16). The median NfL level is indicated by a horizontal line. Statistical analysis was carried out using a two-ANOVA model with interaction. **b** Correlation analysis between SDF-1 levels and Loes score (Spearman's *r*=-0.52; *p*=0.126). **c** Correlation analysis between SDF-1 levels and NfL (Spearman's *r*=-0.15 partialized for sample type (serum vs. plasma); *p*=0.693). Source data are provided as a Source Data file.

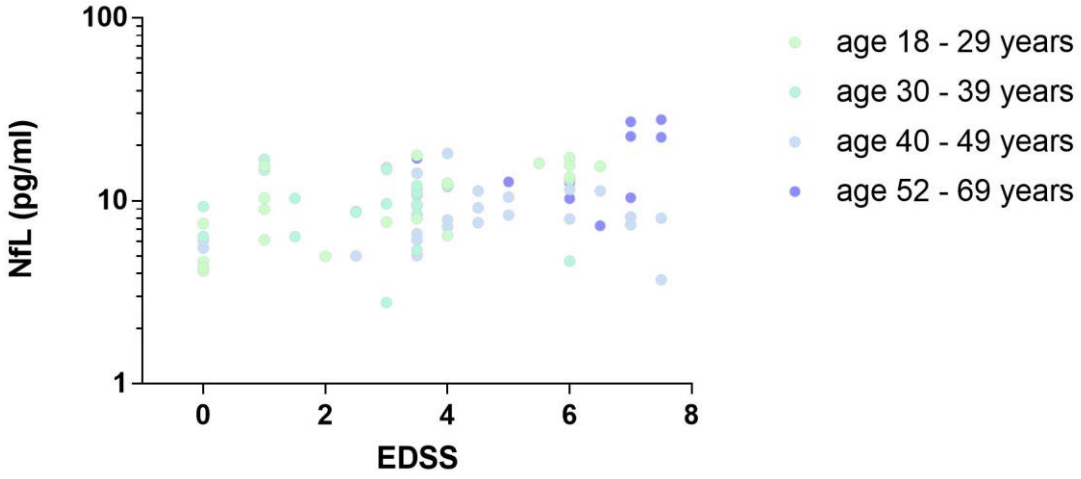

Supplementary Figure 6. Association of EDSS-graded myelopathy and blood NfL levels in AMN patients upon ex-post exclusion of AMN patients that later on developed CALD ("premanifest CALD"). The potential dependence of log of NfL on EDSS-graded clinical severity in individual asymptomatic X-ALD and AMN patients (*n*=58, total sample number=80) with exclusion of patients that developed CALD ("premanifest CALD") during the investigational time of this study was analysed using a linear mixed model with fixed effects EDSS (*p*=0.053) and with age and sample type as adjustment variables (two-sided test). EDSS, Expanded Disability Status Scale. Source data are provided as a Source Data file.

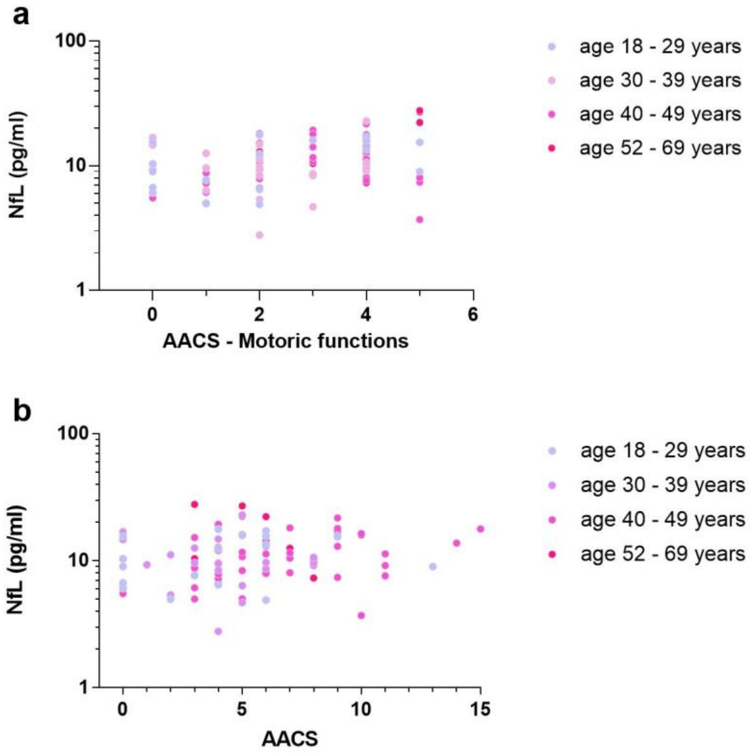

Supplementary Figure 7. Worsening of AACS-graded myelopathy in AMN patients is not reflected by significantly increased NfL. The potential dependence of log of NfL on AACS graded **(a)** motoric and **(b)** combined motoric, bladder, sensory and cerebral functions in individual asymptomatic X-ALD and AMN patients (*n*=60, total sample number including longitudinal samples=93) was analysed using a linear mixed model with fixed effects AACS-motoric function grading or combined AACS grading as well as age and sample type as adjustment variables and a random ID factor. (a) Average increase of 4.6% in NfL levels with each additional AACS-motoric function grading point, *p*=0.246; (b) Average increase of 1.1% in NfL levels with each additional combined AACS grading point, *p*=0.636. AACS, Adulthood ALD/AMN Clinical Scoring System. Source data are provided as a Source Data file.

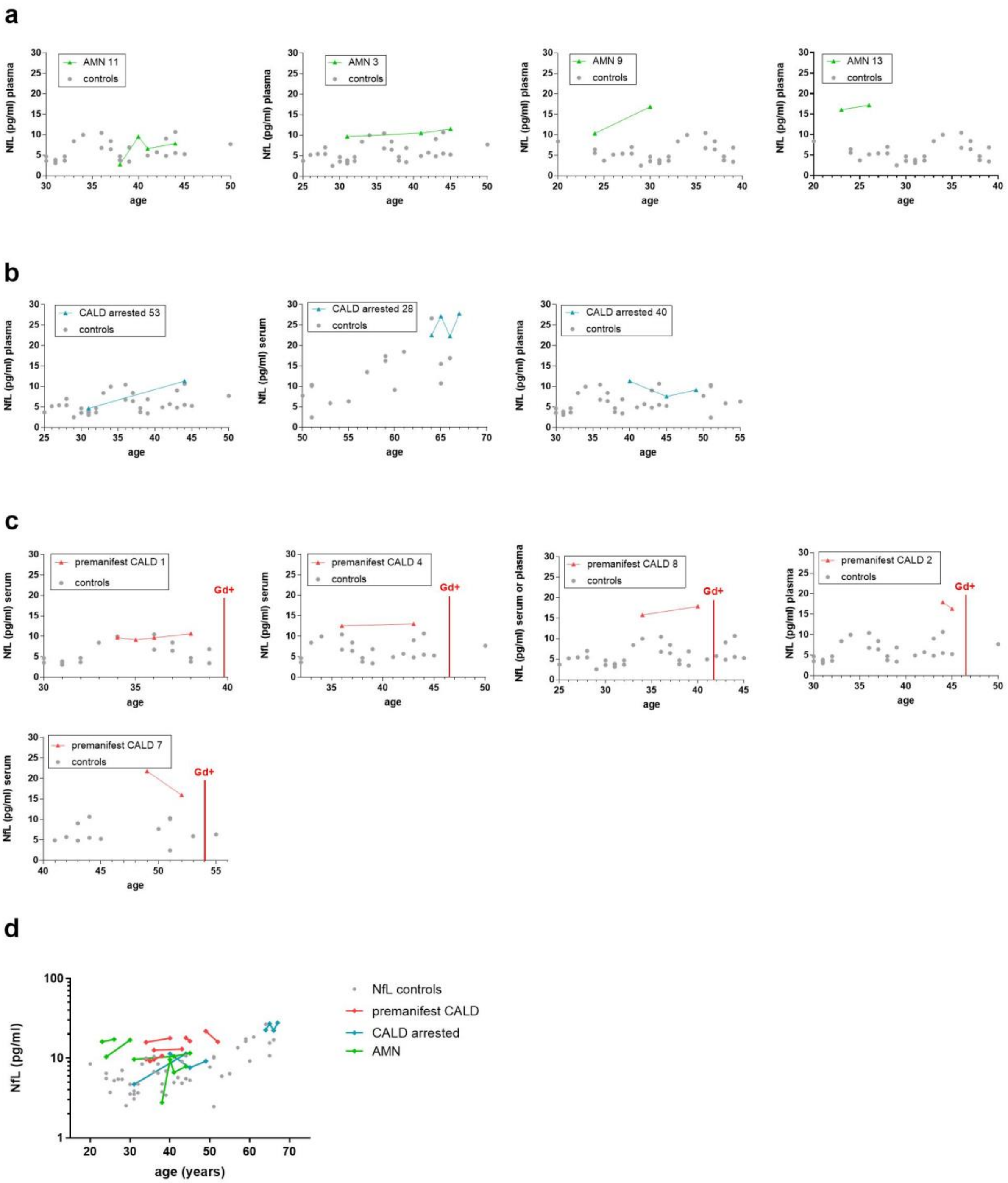

Supplementary Figure 8. Longitudinal assessment of NfL in AMN patients. Follow-up of blood NfL in AMN patients through progression of the myeloneuropathy. **a** AMN patients, **b** AMN patients with self-arrested brain lesions (CALD arrested), **c** AMN patients that at the time of blood sampling were free of inflammatory involvement but later developed CALD, as indicated by presence of Gd-enhancement (Gd+) and **d** longitudinal assessments of NfL levels summarized in one graph. Source data are provided as a Source Data file.

Supplementary Table 1. Detailed characteristics of CALD patients. CALD, childhood onset (age <11 years); CALD, adolescent onset (age 11─ 20 years); CALD, adulthood onset (age ≥21 years).

	**NfL (pg/ml)**	**CALD variant**	**Age (years)**	**MRI brain lesion severity Loes score***
**CALD1**	17.1	adult CALD (smouldering)	48	3.0
**CALD2**	11.1	adult CALD (smouldering)	54	5.0
**CALD3**	22.0	adult CALD (smouldering)	53	18.0
**CALD4**	128.7	adult CALD	43	10.0
**CALD5**	60.4	adult CALD	34	3.0
**CALD6**	13.5<br>28.6	adult CALD	23<br>25	2.0<br>3.0
**CALD7**	52.4	adult CALD	46	3.0
**CALD8**	71.6<br>91.1	adult CALD	51<br>52	4.0<br>6.0
**CALD9**	55.6	adult CALD	31	4.0
**CALD10**	344.1	adult CALD	44	10.0
**CALD11**	312.4	adult CALD	28	10.0
**CALD12**	902.4<br>992.3	adolescent CALD	12<br>12	11.0<br>14.0
**CALD13**	469.7<br>923.9	childhood CALD	11<br>12	10.0<br>13.0
**CALD14**	278.3<br>177.6	adolescent CALD	19<br>20	11.0<br>17.0
**CALD15**	1030.1	adolescent CALD	12	11.0
**CALD16**	142.1<br>294.4	adolescent CALD	16<br>17	14.0<br>19.0
**CALD17**	158.8<br>658.3	adolescent CALD	14<br>15	10.0<br>16.0
**CALD18**	1644.0<br>2066.8	adolescent CALD	11<br>12	10.0<br>16.0
**CALD19**	236.0	childhood CALD	9	8.0
**CALD20**	123.4	childhood CALD	8	18.0
**CALD21**	7.8	childhood CALD	8	2.0
**CALD22**	8.2	adolescent CALD	12	1.0
**CALD23**	119.6	childhood CALD	6	3.5
**CALD24**	340.3	childhood CALD	8	10.0

*Loes score, brain MRI severity scoring system applying a point system ranging from 0 to 34 based on both location and extent of cerebral demyelination as well as atrophy of the brain.

Supplementary Table 2. Detailed characteristics of premanifest CALD patients.

	NfL (pg/ml)	Sample type	Age at sampling (years)	Interval until CALD diagnosis (years)
**Premanifest CALD1**	10.66	serum	38	1.9
**Premanifest CALD2**	16.35	serum	45	1.6
**Premanifest CALD3**	13.79	plasma	43	13
**Premanifest CALD4**	13.03	serum	43	3.4
**Premanifest CALD5**	9.01	plasma	21	3.5
**Premanifest CALD6**	15.89	plasma	40	10.4
**Premanifest CALD7**	15.98	serum	52	2
**Premanifest CALD8**	17.85	plasma	40	1.8
**Premanifest CALD9**	6.7	plasma	18	11.9
**Premanifest CALD10**	22.96	plasma	37	7.4

Supplementary Table 3. NfL in childhood/adolescent and adult CALD patients upon HSCT treatment or spontaneous arrest of brain lesions.

Patient ID	NfL (pg/ml)	Age at sampling (years)	Duration post HSCT (years)	Loes score (at sampling)
CALD post HSCT 1 (=CALD6)	13.7	33	7.1	4
CALD post HSCT 2 (=CALD9)	112.9<br>23.6	33<br>35	1.1<br>3.1	8<br>8
CALD post HSCT 3	132.8	10	0.6	3
CALD post HSCT 4	9.3	19	5.1	15
CALD post HSCT 5	19.3	5	1	3
CALD post HSCT 6	13.9	16	5.5	17
CALD post HSCT 7	27.7	36	9.4	5
CALD arrested 1	5.7	16	-	1
CALD arrested 2	7.5	16	-	1
CALD arrested 28	10.4<br>22.5<br>27.1<br>22.3<br>27.8	57<br>64<br>65<br>66<br>67	-	4<br>4<br>4<br>4<br>4
CALD arrested 40	11.3<br>7.6<br>9.2	40<br>45<br>49	-	12<br>12<br>12
CALD arrested 48	10.7	43	-	5
CALD arrested 51	6.4	33	-	3
CALD arrested 52	8.1	47	-	4
CALD arrested 53	4.7<br>11.4	31<br>44	-	4<br>4
CALD arrested 54	6.5	28	-	3
CALD arrested 58	14.7	38	-	n.d.
CALD arrested 61	13.1	39	-	7
CALD arrested 62	12.2	36	-	2

Supplementary Table 4. The Adulthood ALD/AMN Clinical Scoring system (AACS) [a]

Motor functions (total up to 6)

Score	Degree of disabilty	(Please fill in date at appearance)		Remarks
0	**normal**	Y	N	
0,5	**minimal disability leading to few restrictions in life style but does not interfere with normal life** (description: pt. noticed first or intermittent symptoms, e.g. stiffness in legs, gait disturbances, stumble)			Symptoms may be noticed by a caregiver only
1	**mild disability leading to limited restrictions in normal life* or walking limited (but > 500 m without resting) or positive Romberg testing** (but no increased sway with eyes open, increasing sway with eyes closed but without falling) (*description: first symptoms with frequent or constant stiffness, stumble or balance problems clearly visible for pt. and/or others. Minimal leg weakness may be present, walking distance may be limited, running/jumping may not be possible like previously, e.g. inability to make his previous sport)			Provide date
2	**moderate disability leading to significant restrictions in normal life. Walking limited to 500 m without resting or positive Romberg testing** (increased sway with eyes open already and increasing with eyes closed but without falling)			Sometimes difficult to decide retrospectively
3	**moderate disability leading to significant restrictions in normal life. Walking limited to 100 m without resting or severe gait ataxia** (walking with eyes closed not possible) **or severe sway in Romberg testing** (not possible with eyes closed)			Sometimes difficult to decide retrospectively
4	**walking requiring cane, crutches or walking limited to 50 m without resting**			Provide date
4,5	**walking requiring cane, crutches or walking limited to 20 m without resting**			Sometimes difficult to decide retrospectively
5	**wheelchair bound and/or need of constant help to walk a few steps**			Provide date
6	**inability to stand up and/or sit without help; has some effective use of the arms; bedridden but can communicate and eat**			Provide date

Bladder functions (total up to 3)

Score		(Please fill in date at appearance)		remarks
0	**normal**	Y	N	
1	**Mild urinary or bowel hesitancy or urgency** (description: symptoms are reported by patient and/or caregiver. Single accidents may have occurred. Medications may have been initiated)			Provide date
2	**infrequent urinary or bowel incontinence** (description: several accidents have occurred and/or on medications)			Provide date
3	**Loss of bladder function, complete dependency on catheterization**			Provide date

Sensory symptoms or pain in the legs (total up to 3)

Score		(Please fill in date at appearance)		remarks
0	**Normal sensation, no pain**	Y	N	
1	**mild decrease in sensation, could be intermittent** (any quality, may be up to knee) **and/or pain that is responsive to medications**			Sometimes difficult to decide retrospectively
2	**marked decrease in sensation** (any quality, may be up to hip, needs to be constant) **and/or pain recalcitrant to medication**			Provide date
3	**loss of sensation in legs or decrease sensation below the head**			Provide date

Cerebral functions (total up to 12)

Score		(Please fill in date at appearance)		remarks
0	**normal**	Y	N	
3	**mild or intermittent mood changes, mild behavioral abnormalities, alcohol or other drug problems but no restriction in normal professional life**			Sometimes difficult to decide retrospectively
6	**Moderate but consistent impairment of cortical functions recognized by relatives and/or caregivers, at work or by abnormal neuropsychological testing with restrictions but otherwise continued professional activity** (removal to a lower level or changes to a more simple work may be present but remaining employed)			Provide date
9	**severe impairment of cortical functions incompatible with any professional life and that requires supervision or (discontinuous) assistance in daily life**			Provide date
12	**dementia with loss of all intellectual functions requiring constant supervision or assistance.**			Sometimes difficult to decide retrospectively

Total Adulthood ALD/AMN Clinical Score (AACS) / 24 points

[a] Based on and modified from Köhler et al., "A new disease-specific scoring system for adult phenotypes of X-linked adrenoleukodystrophy", J Mol Neurosci 13, 247-252 (1999) [1]

Supplementary References

1. Köhler, W.S., Sokolowski, M. A new disease-specific scoring system for adult phenotypes of X-linked adrenoleukodystrophy. *Journal of Molecular Neuroscience* **13**, 247-252 (1999).
