# Supplementary material for: Neurofilament light chain as a potential biomarker for monitoring neurodegeneration in X-linked adrenoleukodystrophy
Source: Nat Commun. 2021 Mar 22;12:1816. doi: 10.1038/s41467-021-22114-2 (PMC7985512; doi:10.1038/s41467-021-22114-2)
Supplement: Supplementary file 3 — Reporting Summary [file 41467_2021_22114_MOESM3_ESM.pdf]

nature research

Corresponding author(s): Johannes Berger

Last updated by author(s): 14/01/21

Reporting Summary

Nature Research wishes to improve the reproducibility of the work that we publish. This form provides structure for consistency and transparency in reporting. For further information on Nature Research policies, see our Editorial Policies and the Editorial Policy Checklist.

Statistics

For all statistical analyses, confirm that the following items are present in the figure legend, table legend, main text, or Methods section.

n/a	Confirmed	
☐	☒	The exact sample size (n) for each experimental group/condition, given as a discrete number and unit of measurement
☐	☒	A statement on whether measurements were taken from distinct samples or whether the same sample was measured repeatedly
☐	☒	The statistical test(s) used AND whether they are one- or two-sided<br>*Only common tests should be described solely by name; describe more complex techniques in the Methods section.*
☐	☒	A description of all covariates tested
☐	☒	A description of any assumptions or corrections, such as tests of normality and adjustment for multiple comparisons
☐	☒	A full description of the statistical parameters including central tendency (e.g. means) or other basic estimates (e.g. regression coefficient) AND variation (e.g. standard deviation) or associated estimates of uncertainty (e.g. confidence intervals)
☐	☒	For null hypothesis testing, the test statistic (e.g. F, t, r) with confidence intervals, effect sizes, degrees of freedom and P value noted<br>*Give P values as exact values whenever suitable.*
☒	☐	For Bayesian analysis, information on the choice of priors and Markov chain Monte Carlo settings
☒	☐	For hierarchical and complex designs, identification of the appropriate level for tests and full reporting of outcomes
☒	☐	Estimates of effect sizes (e.g. Cohen's d, Pearson's r), indicating how they were calculated

Our web collection on statistics for biologists contains articles on many of the points above.

Software and code

Policy information about availability of computer code

Data collection	Microsoft Excel 2016,IBM SPSS Version 21
Data analysis	SAS 9.4; GraphPad Prism 7.00, nQuery (version 8)

For manuscripts utilizing custom algorithms or software that are central to the research but not yet described in published literature, software must be made available to editors and reviewers. We strongly encourage code deposition in a community repository (e.g. GitHub). See the Nature Research guidelines for submitting code & software for further information.

Data

Policy information about availability of data

All manuscripts must include a data availability statement. This statement should provide the following information, where applicable:
- Accession codes, unique identifiers, or web links for publicly available datasets
- A list of figures that have associated raw data
- A description of any restrictions on data availability

All data are included in the manuscript or in the Supplementary Materials and is available from the corresponding author on reasonable request.

Field-specific reporting

Life sciences study design

All studies must disclose on these points even when the disclosure is negative.

Sample size	Based on preliminary experiments, we performed sample size calculations for both investing AMN and CALD using the nQuery software (version 8). For the comparison AMN vs. healthy controls, the sample size calculation was performed after log-transforming NfL levels with base 1.35. This calculation revealed that a sample size of 37 in each group will have 85% power to detect a difference in means of 1.0 on the log 1.35 scale (corresponding to 35% increase in geometric means in the original scale) assuming that the common standard deviation is 1.4 (based on pooling the observed standard deviations on the log 1.35 scale in the two groups from the preliminary data) using a two group t-test with a 5% two-sided significant level. For CALD, where the minimal clinically relevant difference was set to 70%, the power calculation revealed that this could be detected with a power of 85% with 13 patients per group using a two group t-test with a 5% two-sided significance, based on a pooled standard deviation estimate from the preliminary data on the log-scale as above.
Data exclusions	No data were excluded.
Replication	The SiMoA measurements of a limited sample set (n=11) were successfully reproduced by measuring these samples again at a later time point at another institution by a different investigator.
Randomization	Patients were not randomized. Samples from participants were allocated into different groups based on predefined inclusion and exclusion criteria. For the asymptomatic X-ALD (n=7) and AMN (n=61) cohorts, we included participants that presented without (asymptomatic X-ALD) or with symptoms of AMN according to EDSS, lacked acute inflammatory CALD brain lesions on MRI, had not received HSCT or gene therapy, had no other neurological disease interfering with the assessment of myelopathy, and had not participated in a clinical trial within the last year before blood sampling. CALD patients (n=24) with Gd-enhancing brain MRI lesions at the time of blood sampling and clinical progression graded by the MRI severity score of Loes 17 were included if they had not undergone HSCT and had not been in enrolled in clinical trials within the last year before blood sampling. The HSCT group (n=7) consisted of participants that met the other inclusion criteria for CALD patients but received HSCT resulting in the halt of inflammatory brain lesions as established by the absence of Gd-enhancement on MRI. The childhood/adolescent CALD self-arrested set consisted of samples from two patients in which CALD inflammatory brain lesions spontaneously self-arrested in childhood/adolescence and that were asymptomatic for AMN symptoms. The healthy control cohort consisted of 48 adult and 7 childhood/adolescent male participants with no diagnosis of disorders characterized by neuroinflammation and/or axonal degeneration, such as X-ALD, amyotrophic lateral sclerosis, multiple sclerosis or any dementia.
Blinding	All samples were pseudonymized and the NfL measurement was carried out blinded for the phenotype and genotype of donors.

Reporting for specific materials, systems and methods

We require information from authors about some types of materials, experimental systems and methods used in many studies. Here, indicate whether each material, system or method listed is relevant to your study. If you are not sure if a list item applies to your research, read the appropriate section before selecting a response.

Materials & experimental systems

n/a	Involved in the study	
☒	☐	Antibodies
☒	☐	Eukaryotic cell lines
☒	☐	Palaeontology and archaeology
☒	☐	Animals and other organisms
☐	☒	Human research participants
☒	☐	Clinical data
☒	☐	Dual use research of concern

Methods

n/a	Involved in the study	
☒	☐	ChIP-seq
☒	☐	Flow cytometry
☐	☒	MRI-based neuroimaging

Human research participants

Policy information about studies involving human research participants

Population characteristics	The covariate-relevant population characteristics are for the childhood/adolescent control group: male, median age 11 (10-12), no diagnosis of disorders characterize by neuroinflammation and/or axonal degeneration. Adult controls: male, mean age 39 (31-52), no diagnosis of disorders characterize by neuroinflammation and/or axonal degeneration. Asymptomatic X-ALD: male, mean age = 31 (26 - 40), diagnosed with X-ALD based on VLCFA accumulation, no signs of myelopathy, absence of neuroinflammation. AMN: male, median age = 40 (31-45), diagnosed with X-ALD based on VLCFA accumulation, symptoms of AMN according to EDSS or AACS grading, absence of Loes-score graded neuroinflammation. Childhood/adolescent CALD: Male, median age = 12 (9-15), diagnosed with X-ALD based on VLCFA accumulation, Loes-score graded neuroinflammation. Adult CALD: Male, median age = 44 (30-52), diagnosed with X-ALD based on VLCFA accumulation, Loes-score graded neuroinflammation. Self-arrested childhood/adolescent CALD: male, median age = 16 (16-16), diagnosed with X-ALD based on VLCFA accumulation, previous Loes-score graded neuroinflammation that self-arrested at the time of sampling (no Gd-enhancement in MRI). CALD post HSCT: male, mean age = 26 (12-35), diagnosed with X-ALD based on VLCFA accumulation, past diagnosis with Loes-score graded CALD, previous HSCT at time of sampling. The population characteristics are described in Table 1 and Tables S1,S2 and S3.
Recruitment	Blood samples from X-ALD patients and healthy controls were derived from routine diagnostic tests at the clinics. Thus, no active recruitment took place and all available samples without self-selection were included in the analysis.
Ethics oversight	Ethical aspects were approved by the Ethical Review Board at the Medical University of Vienna (EK Nr: 1613/2019)

Note that full information on the approval of the study protocol must also be provided in the manuscript.

Magnetic resonance imaging

Experimental design

Design type	n/a
Design specifications	n/a
Behavioral performance measures	n/a

Acquisition

Imaging type(s)	structural
Field strength	1.5 Tesla
Sequence & imaging parameters	Fig. 3 A, B/2, C/2, D/2, E/1: T2 FLAIR, axial 3mm, FoV 230x230, matrix 256X230, TE 99, TR 8000; Fig. B/1, C/1, D/1, E/1: T1 SE, axial 3mm, FoV 129x230, matrix 320x168, TE 14, TR 854
Area of acquisition	whole brain

Diffusion MRI ☐ Used ☒ Not used

Preprocessing

Preprocessing software	n/a
Normalization	n/a
Normalization template	n/a
Noise and artifact removal	n/a
Volume censoring	n/a

Statistical modeling & inference

Model type and settings	n/a
Effect(s) tested	n/a

Specify type of analysis: ☒ Whole brain ☐ ROI-based ☐ Both

Statistic type for inference (See Eklund et al. 2016)	voxel-wise
Correction	n/a

Models & analysis

n/a | Involved in the study

- [x] [ ] Functional and/or effective connectivity
- [x] [ ] Graph analysis
- [x] [ ] Multivariate modeling or predictive analysis
